# Optimal SSSC-based power damping inter-area oscillations using firefly and harmony search algorithms

**DOI:** 10.1038/s41598-020-69123-7

**Published:** 2020-07-22

**Authors:** Amirreza Naderipour, Zulkurnain Abdul-Malek, Vigna K. Ramachandaramurthy, Mohammad Reza Miveh, Mohammad Jafar Hadidian Moghaddam, Josep. M. Guerrero

**Affiliations:** 1 Institute of Research and Development, Duy Tan University, Da Nang, 550000 Vietnam; 2 Faculty of Electrical – Electronic Engineering, Duy Tan University, Da Nang, 550000 Vietnam; 3 Institute of High Voltage & High Current, School of Electrical Engineering, Faculty of Engineering, Universiti Teknologi Malaysia, 81310 Johor Bahru, Malaysia; 4 Institute of Power Engineering, Department of Electrical Power Engineering, College of Engineering, Universiti Tenaga Nasional, Jalan Ikram-Uniten, 43000 Kajang, Malaysia; 5 Department of Electrical Engineering, Tafresh University, 39518-79611 Tafresh, Iran; 6 College of Engineering and Science, Victoria University, Melbourne, 3047 Australia; 7 Institute of Energy Technology, Aalborg University, Aalborg East, Alboorg, Denmark

**Keywords:** Energy science and technology, Engineering

## Abstract

The static synchronous series compensator (SSSC) can add a series reactance to the transmission line, and when it is fed using auxiliary signals, it can participate in damping inter-area oscillations by changing the series reactance. In this paper, the effect of the SSSC on small-signal stability is investigated. The design of a controller for damping oscillations is designed and discussed. Moreover, using the firefly and the harmony search algorithms, the optimal parameters controlling SSSC are addressed. The effectiveness of these two algorithms and the rate of SSSC participation in damping inter-area oscillation are also discussed. MATLAB software was used to analyse the models and to perform simulations in the time domain. The simulation results on the sample system, in two areas, indicated the optimal accuracy and precision of the proposed controller.

## Introduction

Inter-area oscillations are common phenomena in the interconnected power systems; therefore, damping of these oscillations have become one of the main difficulties in the power system. To date, this issue has been addressed by the power system stabiliser (PSS). However, the conventional PSS is based on local signals and cannot effectively dampen inter-area oscillations that occur within the power system; therefore, adaptive and robust methods for designing PSS are needed^1^. On the other hand, the use of flexible alternating current transmission systems (FACTS), which can control the impedance of the power flow on transmission lines and the bus voltage, is a novel solution^2^. Among different series power electronics-based FACTS controllers, the static synchronous series compensator (SSSC) is one of the best alternative means to improve power oscillation damping. It is based on a voltage source converter connected to a transmission line in series, and it adds the sinusoidal voltage along the q axis into the line current. Thus, the SSSC can use a series reactance with a transmission line, and this reactance can be inductive or capacitive. Using auxiliary signals in the SSSC controller can improve the damping of inter-area oscillations by altering the impedance of the transmission line. In Wang and Du^3^, an ideal controller is assumed, and the SSSC is used to damp small-signal oscillations based on the Phillips–Heffron model. In Darabian and Jalilvan^4^, the SSSC risk model was applied to use a conventional PSS power stabiliser to eliminate oscillations. In Norouzi and Sharaf^5^, two voltage regulation strategies were compared with the SSSC, and Kumar and Ghosh^6^ proposed a controlling structure for SSSCs. In Bongiorno et al. (2008)^7^, the structure proposed in Kumar and Ghosh^6^ was used, and instead of changing the rotor speed as a control signal, transmission line power variations were used to detect the presence of high influences in wind farms. In Shen et al.^8^, an adaptive neural controller was used to improve the control of damping small-signal oscillations with SSSC.

In Xia et al.^9^, suggested a damping control design for an interline power flow controller (IPFC) comprise two voltage sourced converters (VSCs) to maximize voltage-stability limited power transfer and damp power swings. In another major study, Xia et al.^10^, offered a new computation method to regulate the dispatchable control modes of the convertible static compensator (CSC) in a power system. Xia et al.^11^, proposed a stability improvement using VSCs based FACTS device. The paper shows that the FACTS devices have the ability to increase the transient power transfer capability of a transmission system under abnormal conditions.

Xinghao et al.^12^, proposed two sensitivity approaches including an injected voltage source formulation and the equivalent impedance formulation in the dispatch and placement of FACTS devices. In Aranya^13^, to mitigate interarea oscillation in large power systems, wide-area damping control using dynamic clustering and TCSC-based redesigns is presented. The method involved coherent clustering using Synchrophasors and designing controllers to attain preferred damping between the clusters. The suggested controller contains three steps. Model reduction, aggregate control and control inversion are these three steps.

In this paper, the effect of SSSC on small-signal stability of a multi-machine power system is investigated, and a control structure is proposed for the SSSC compensator. Modal analysis and time-domain simulation are done on a two-area sample system. Finally, the system, which is located in two areas, is simulated with optimised stabilisers by harmony search and firefly algorithms, and the ability of these two optimisation methods to improve small-signal stability is evaluated.

The remainder of this article is arranged as follows: Part II is dedicated to the SSSC voltage regulator. The effects of the SSSC on the small-signal stability of the power system and the proposed controller are presented in “SSSC effect on small-signal stability” and “SSSC damping controller”, respectively. In “Intelligent random optimisation algorithms”, i.e., the firefly and harmony search algorithms^14^, are introduced. In “two-area system”, optimisation of the proposed controller parameters is performed, and the simulation results on sample systems are shown in “Optimising SSSC parameters”. The final section presents our conclusions.

## Methods

### SSSC modeling

**Figure 1:**
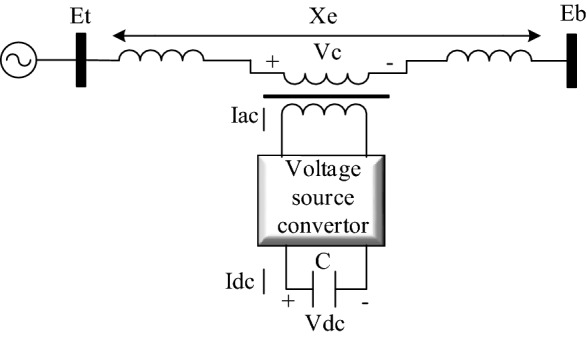
Single-machine infinite-bus system with SSSC^17^.

In Fig. 1, a single-machine infinite bus system with SSSC is presented. The voltage generated by the SSSC is controlled by the pulse-width modulation (PWM) method to ensure that the voltage is always in line with the *q* axis relative to the line current^15^. Therefore, the SSSC can act as a capacitor or inductor in the system. The magnitude of the PWM controls the degree of compensation, and the phase angle determines the type of compensation: capacitive or inductive^16^.

**Figure 2:**
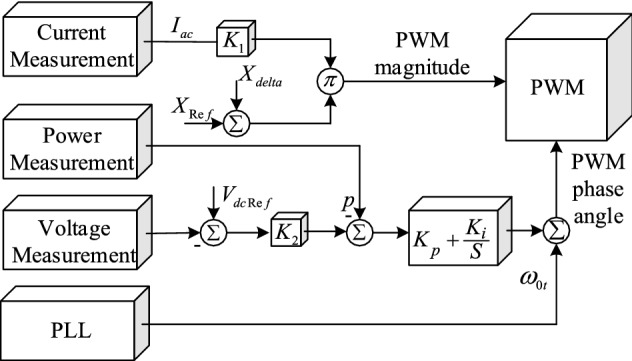
SSSC voltage regulation diagram^17^.

The PWM voltage-generation diagram is shown in Fig. 2. The magnitude of the PWM signal is obtained from multiplying the SSSC-measured AC by the AC generated in the reference reactance. The *X*_*sdelta*_ is calculated using the proposed controller in the fifth section. The phase angle of the PWM is obtained by the synchronous phase angle, and a proportional-integral (PI) controller keeps the DC voltage constant so that the active power exchange between the power system and the SSSC is zero. As a result, the phase angle generated by the diagram in Fig. 2 ensures that the voltage phasor and flow phasor of the transmission line are perpendicular to each other and that the SSSC behaves like a reactance.

### SSSC effect on small-signal stability

The ability of the power system to maintain the synchronism of synchronous generators during small disturbances is called small-signal stability^18^. The single-machine infinite-bus system in Fig. 1 is considered for the analysis of small-signal stability in which the dynamics of the synchronous generator stator are ignored^8^. Moreover, to maintain the simplicity, the dampers and resistances of all synchronous generator rotors are ignored. As a result, only a differential equation for the excitation circuit voltage plus two differential equations for the rotational motion of the synchronous generator remain, as expressed in (1).

1 $$\begin{aligned} p\omega_{r} & = \frac{1}{2H}\left( {T_{m} - T_{e} - K_{{D\omega_{r} }} } \right) \\ p\delta & = \omega_{o} \omega_{r} \\ p\Psi_{fd} & = \frac{{\omega_{o} R_{fd} }}{{L_{adu} }}E_{fd} - \omega_{o} R_{fd} i_{fd} \\ \end{aligned}$$

where $p$ is the first-order derivation, $\Delta \omega_{r}$ is rotor speed variations, $H$ is inertial constant, ${T}_{m}$ is mechanical torque, ${T}_{e}$ is electromagnetic torque, ${K}_{D}$ is mechanical damping coefficient, $\delta$ is rotor angle, ${\omega }_{0}$ is rated turbine speed, ${\Psi }_{fd}$ is excitation circuit’s flux linkage, ${R}_{fd}$ is excitation circuit’s resistance, ${E}_{fd}$ is the excitation output voltage, ${L}_{adu}$ is non-saturation mutual inductance between the rotor and axis d of the stator and ${i}_{fd}$ is Excitation current.

For a full presentation of the state model, ${T}_{e}$ and ${i}_{fd}$ must be expressed in terms of the state variables. For electromagnetic torque:

2 $$i_{fd} = \frac{{\Psi_{aq} - \Psi_{ad} }}{{L_{fd} }}$$

where ${\Psi }_{ad}$ and ${\Psi }_{aq}$ are the flux linkage components between the stator and the rotor on the *d* and *q* axes, respectively and ${i}_{d}$ and ${i}_{q}$ are stator currents. The equation for the flux linkage of the excitation circuit is:

3 $$\begin{aligned} \Psi_{ad} & = - L_{ads} i_{d} + L_{ads} i_{fd} \\ \Psi_{aq} & = - L_{aqs} i_{q} \\ \end{aligned}$$

where ${L}_{fd}$ is the self-inductance of the excitation circuit. Mutual flux linkage relations are also expressed in Eq. (4) such that ${L}_{ads}$ is the interactive saturation inductance between the rotor and stator.

4 $$\begin{aligned} \Psi_{ad} & = - L_{ads} i_{d} + L_{ads} i_{fd} \\ \Psi_{aq} & = - L_{aqs} i_{q} \\ \end{aligned}$$

Stator voltage equations are also described in (5), where $e_{d}$ and $e_{q}$ are the voltage components of generator’s terminal on the *d* and *q* axes, respectively. For the terminal voltage in the rotor synchronous reference frame:

5 $$\left. \begin{aligned} e_{d} & = \, L_{l} i_{q} - \Psi_{aq} \\ e_{q} & = - L_{l} i_{d} + \Psi_{ad} \\ \end{aligned} \right\} \Rightarrow E_{t} = e_{q} + je_{d}$$

where $L$ is the stator leakage inductance. Because of how the turbine is connected to the infinite bus, the following relations govern:

6 $$\begin{aligned} e_{d} & = - {\text{X}}_{E} i_{q} + e_{bd} \\ e_{q} & = {\text{ X}}_{E} i_{d} + e_{bq} \\ \end{aligned}$$

**Figure 3:**
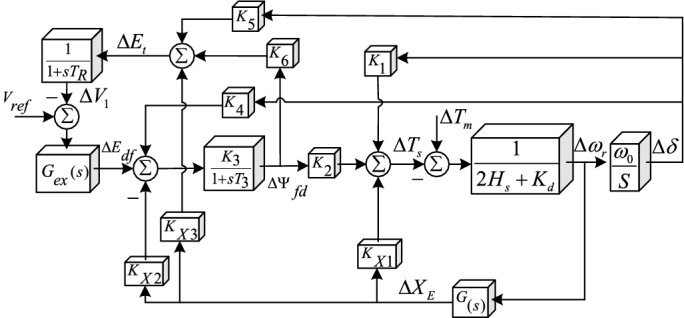
Block diagram of the SMIB system.

The variable ${X}_{E}$ is the equivalent reactance of the transmission line, and ${e}_{bd}$ and ${e}_{bq}$ are infinite bus voltage components. To analyse the small signal, the state equations of the system must be linearised. According to the method presented in^16^ and considering the effect of the SSSC on the small-signal stability, the variable $\Delta {X}_{E}$, which represents the change in the transmission line reactance, is defined in the linearization of the system. After the linearization of relations (5) and (6); rewriting the relations (2), (3) and (4); and inserting the block of the small-signal stability into (6); the single-machine infinite-bus (SMIB) system is obtained as presented in Fig. 3.

In Fig. 3, the conversion function excitation system between ${G}_{ex}(s)$ and $G(s)$ is the conversion function of the SSSC damper controller. The *K* constants are defined as ${k}_{1}$ to ${k}_{6}$ as in 8. If SSSC is responsive to rapid changes in ${X}_{delta}$, then the red lines in the block diagram of the system indicate the impact of the SSSC. Coefficients ${K}_{X1}$, ${K}_{X2}$, and ${K}_{X3}$ include:

7 $$\begin{aligned} K_{X1} & = (n_{3} + n_{4} )(\psi_{ado} + L_{aqs} i_{do} ) - (m_{3} + m_{4} )(L_{ads} i_{qo} + \psi_{aqo} ) \\ K_{X2} & = \frac{{L_{adu} \, L_{ads} }}{{L_{fd} }}(m_{3} + m_{4} ) \\ K_{X3} & = \frac{{e_{do} }}{{E_{to} }}(L_{l} + L_{aqs} )(n_{3} + n_{4} ) - \frac{{e_{qo} }}{{E_{to} }}(L_{l} + L_{ads} )(m_{3} + m_{4} ) \\ \end{aligned}$$

and the coefficients ${n}_{3}$, ${n}_{4}$, ${m}_{3}$, and ${m}_{4}$ include:

8 $$\begin{aligned} n_{3} & = \frac{{e_{bdo} }}{D} \\ m_{3} & = \frac{{\Psi_{fdo} \frac{{L_{ads} }}{{L_{ads} + L_{fd} }} - e_{bqo} }}{D} \\ m_{4} & = \frac{{ - (L_{l} + X_{Eo} + L_{aqs} )(2X_{Eo} + L_{aqs} + L_{ads}^{^{\prime}} )m_{3} }}{D} \\ n_{4} & = \frac{{(L_{l} + X_{Eo} + L_{aqs} )(2X_{Eo} + L_{aqs} + L_{ads}^{^{\prime}} )n_{3} }}{D} \\ \end{aligned}$$

The participation of the controller of the SSSC compensator in increasing system damping occurs through the blocks ${K}_{X1}$, ${K}_{X2}$, and ${K}_{X3}$. As shown in Fig. 3, the participation of ${K}_{X1}$ is direct, but that of ${K}_{X2}$ and ${K}_{X3}$ is indirect through the first-order post-phase blocks. Based on reference^2^, the effects of ${K}_{X2}$ and ${K}_{X3}$ are negligible relative to ${K}_{X1}$, and when the SSSC damper controller is designed, the *G(s)* function adds a zero- or 180-degree phase shift, depending on the sign of ${K}_{X1}$. For multimachine systems, relations (1) to (6) are transformed into matrix relationships and constants *K* are converted into a matrix or vector with the corresponding variables in different machines. The diagram in Fig. 3 remains valid, but for each block matrix, the variables between the turbines are coupled.

### SSSC damping controller

**Figure 4:**
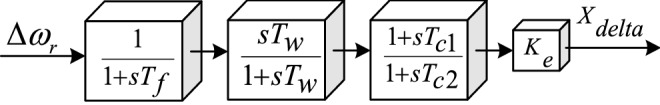
SSSC damping controller diagram.

The SSSC damping controller is shown in Fig. 4. The low pass filter for removing high frequencies from variations in the signal uses the speed of the generator. The frequency range in small-signal stability is from 0.1 to 2 Hz; therefore, the cutoff frequency of the low-pass filter is considered as 10 Hz.

The washout filter also acts as a high-pass filter, which allows the high-frequency oscillations to pass through but eliminates the stable state and removes the damping blocks in the steady state. Therefore, these two filters ensure that the controller only responds to the frequencies in the studied range of small-signal stability.

### Intelligent random optimisation algorithms

Optimisation, random search, and evolutionary algorithms are new and efficient methods to find optimal solutions to problems^19^. The randomness of these algorithms prevents them from being trapped in local optima. In practical optimisation problems, such as engineering design, organisational management, and economic systems, the focus is on obtaining optimal and general solutions. Many of these algorithms are inspired by biological systems; the firefly algorithm (FA) is one example.

#### Firefly algorithm

The FA was presented in 2005, and the theoretical bases for this algorithm were developed in 2006–2008^20^. This algorithm searches for an optimal solution to the problem by modelling the behaviour of a set of fireflies. FA allocates values related to the location fitness of each firefly as a model for firefly pigments and updates their location in successive repetitions of the algorithm^21^. The two main phases of the algorithm in each replication are updating the pigment and motion. Fireflies move to other fireflies in their vicinity that have more pigment. Accordingly, during successive iterations, the set tends to provide a better answer.

Mass intelligence, as it occurs in natural communities, is the result of actions that are carried out by individuals according to local information. Typically, the behaviour of the masses leads to more complex and massive targets. Examples of this phenomenon include ants, honeybees, birds, etc. The decentralised decision-making mechanisms in these and other natural species inspired the design of large-scale algorithms for solving complex problems such as optimisation, multi-criteria decision making, and robotics. In this section, an algorithm based on the firefly social behaviour has been investigated.

#### Initialising the fireflies

The FA is initiated by randomly placing an n-member population of fireflies at different points in the search space. Initially, all fireflies have the same amount of luciferin as member 1. Random values must first be selected for the independent variables of the problem. Each replication of the algorithm includes an update phase for luciferin and another for fireflies.

#### Updating luciferin

The amount of luciferin in each firefly is determined during each iteration, depending on the fitting of its location. Thus, during each iteration, a value is added to the current luciferin of each firefly according to the amount of fitness determined for that firefly. In addition, to model the gradual decrease of the residual value, the amount of current luciferin is reduced by a factor of less than 1. In this way, the relationship between luciferin updates is as follows:

9 $$\ell_{i} (t) = (1 - \rho )\ell_{i} (t - 1) + \gamma J(x_{i} (t))$$

where $li(t), li(t - 1)$, and $J(xi(t))$ are the new luciferin value, the previous luciferin value, and location fitness of firefly $i$ in repetition $t$ of the algorithm, and $\rho$ and $\gamma$ are fixed numbers for modeling the gradual decline and the effect of fitness on luciferin, respectively. At this stage, the fitness of each member of the population must be calculated. Accordingly, the fitness of each member of the algorithm population is the value of the objective function defined for the problem with the values of the independent variables attributed to the fitted firefly.

#### Firefly motion

During the motion phase, each firefly moves toward one of its neighbours with higher luciferin probabilistically. For each firefly, *i* probabilities of moving to a brighter neighbour *j* are defined as follows:

10 $$p_{ij} (t) = \frac{{\ell_{j} (t) - \ell i(t)}}{{\sum {_{{k \in N_{i} (t)}} } \ell_{k} (t) - \ell_{k} (t)}}$$

where *N*_*i*_(*t*) is the set of fireflies neighbouring firefly $i$ at time $t$, *d*_*ij*_(*t*) is the Euclidean distance between the firefly $i$ and $j$ at time $t$, and *r*_*di*_(*t*) indicates the neighbouring range of the variable related to firefly $i$ at time $t$. Assuming that firefly $j$ is selected by firefly $i$ (with probability $p$), the discrete-time equation of the firefly can be written as follows:

11 $$x_{i} (t + 1) = x_{i} (t) + s\left(\frac{{x_{i} (t) - x_{i} (t)}}{{\left\| {x_{i} (t) - x_{i} (t)} \right\|}}\right)$$

where xi(t) is the m dimension vector of firefly i location at time t, the $\left\| {x_{i} (t) - x_{i} (t)} \right\|$ operator shows the Euclidean norm, and s is the hop size.

#### Updating the neighbourhood range

By assuming ${R}_{o}$ is the initial neighbourhood range for each firefly, the neighbourhood range of each firefly is updated during each iteration of the algorithm as follows:

12 $$r_{i}^{d} (t + 1)\min \left\{ {r_{s} ,max\left\{ {0,r_{i}^{d} (t) + \beta \left( {n_{t} - \left| {N_{i} (t)} \right|} \right)} \right\}} \right\}$$

where $\beta$ is a constant parameter and ${n}_{t}$ is a parameter for controlling the number of neighbours.

#### Stop criterion

If the stop criterion for the algorithm is not met, then the algorithm will perform another iteration. Of course, the stop criterion can be defined as a fixed number of iterations to reduce the speed and precision of the estimation of $K$ variables. Each firefly with the highest fitness value is considered as the output of the algorithm.

It should be noted here that global optimization approaches have regularly shown improper slow convergence rates because of their random search, particularly near the area of the global optimum. The firefly algorithm may not find the actual optimal if it started from a different initial condition. Consequently, it is needed to investigate the effect of this uncertainty in the damping performance. In this regard, the hybrid algorithms can benefit from the advantages of both methodologies and alleviate their inherent disadvantages are of interest. Future studies on the current topic are therefore recommended. Using this approach, during the initial optimization stages, the hybrid algorithm starts with an algorithm to find a near-optimum solution and accelerate the convergence speed. The searching process is then switched to FA and the best solution found by another algorithm will be taken as the initial starting point for the FA and will be fine-tuned. In this way, the hybrid algorithm may find an optimum solution more quickly and accurately.

### Harmony search algorithm

Over the past decade, to overcome the computational deficiencies of mathematical algorithms, evolutionary or metaheuristic algorithms, such as annealing and genetic algorithms, have been invented and simulated. However, searching for more powerful algorithms remains a challenge for engineers. The harmony search (HS) algorithm is a powerful search algorithm for finding the optimal answer^22^.

In composing music, several musicians collaborate with different instruments. Their goal is to produce beautiful music. During this collaborative process, each musician attempts to choose the best music performance each time to create better music. The beauty of music improves during collaboration. Typically, an attempt is made to evolve the music at each stage so that harmony is created between musicians.

Over time, the musicians produce a musical piece by playing different harmonies. After playing several pieces, the musicians recall the pieces they have played (the harmonies of that piece). Suppose that there are $K$ harmonies composed by *n* musicians, and it is assumed that the size of each musicians’ memory (HMS) is equal to $K$ harmony. Therefore, according to the following equation, a matrix with k rows (the number of memorised harmonies) and $n + 1$ columns, in which n is the number of musicians (the number of variables affecting the problem), and a column for the value of that harmony considering the fitness function is considered. The matrix is called the HM, or the harmony memory.

This algorithm consists of five steps:

1. Initialising the optimisation problem and initial parameters
2. Initialising harmony memory
3. Creating a new, improved harmony
4. Updating harmony memory
5. Repeating steps 3 and 4 until the final condition is satisfied or the desired number of iterations is completed
6. Optimising the control parameters of the SSSC damper.

To increase the damping of the studied system, two filters and a pre-phase/post-phase compensator block are located in the designed controller. The coefficients and time constants of the low-pass filter blocks and the washout filters are determined according to the studied frequency domain. Therefore, the cutoff frequency of the low-pass filter is considered as 10 Hz, and the test range in the small-signal stability is 0.2–2 Hz. The time constant of the phase compensator and compensator gain are considered as optimisation parameters. The optimal values of these parameters make it possible for the SSSC damper controller to have the maximum effect in eliminating the low-frequency oscillations of the power system. Thus, the optimisation vector is defined as follows:

13 $$X_{POP} = [K_{e} {\text{ T}}_{1} {\text{ T}}_{2} ]$$

This vector is the input to the optimisation algorithm. A reasonable range for the search space is determined by the change in the variables of the algorithm. By maintaining the integrity of the problem, the following intervals are expressed for the input vector:

14 $$\begin{gathered} {\text{o}} \le T_{C1} \le 1 \hfill \\ {\text{o}} \le T_{C2} \le 1 \hfill \\ 1 \le K_{e} \le 20 \hfill \\ \end{gathered}$$

To increase the damping of the system and reduce inter-area power oscillations, two control signals are considered. The first signal is the rotor angle variation of the generators, and the second signal is the power variation in the inter-area transmission line. Therefore, for each of these control signals, a proportional objective function is defined that ITAE criterion is considered in designing these functions.

15 $$\begin{gathered} OF_{1} = ITAE = \int\limits_{o}^{t} {t\left| {\Delta \omega_{r} } \right|} \, dt \hfill \\ OF_{2} = \int\limits_{o}^{t} {t\left| {\Delta P_{12} } \right|} \, dt \hfill \\ \end{gathered}$$

where ${\Delta P}_{1, 2}$ is the inter-area power oscillation in the transmission line^23^. The purpose of using intelligent algorithms is to minimise the defined objective functions according to the range of variation in the control variables^24^, and minimising each objective function equals the maximum damping of the system and the minimum number of small-signal oscillations.

## Results and discussion

### Two-area system

**Figure 5:**
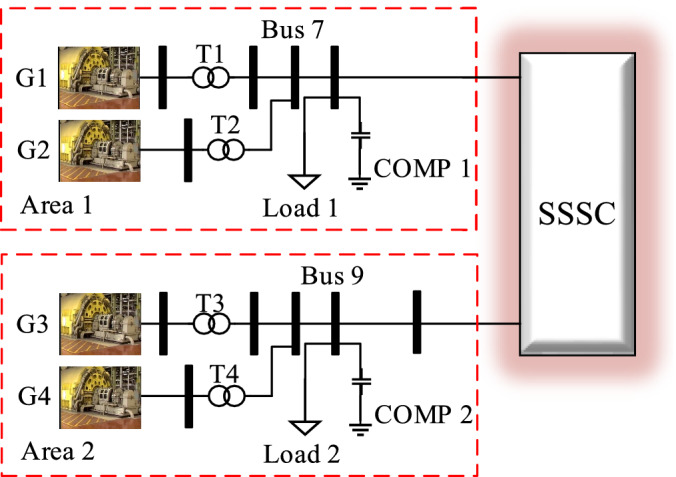
Two-area four-machine system.

To investigate stability in the power system, a two-area power system with SSSC is considered. This system, shown in Fig. 5, is simulated in a Matlab-Simulink software environment. Synchronous generators are modelled as a sixth-order model and the SSSC converter.

The system consists of two similar areas connected by a weak transmission line. Each area consists of two interconnected synchronous generators rated at 900 MVA and 20 kV. The voltage of the transmission line system is rated at 230 kV, and in the normal state without the SSSC, 340 MW of active power is transferred from the first to the second area. Synchronous generator No. 3 is considered as the base. The amount of active power of each synchronous generator and the voltage of turbines are given in Table 1.

**Table 1:** Synchronous generator parameters.

Generator	Active Power	Voltage
G_1_	700 MW	1/03
G_2_	700 MW	1/01 $\angle {\text{o}}$
G_3_	719 MW	1/03
G_4_	700 MW	1/01

**Figure 6:**
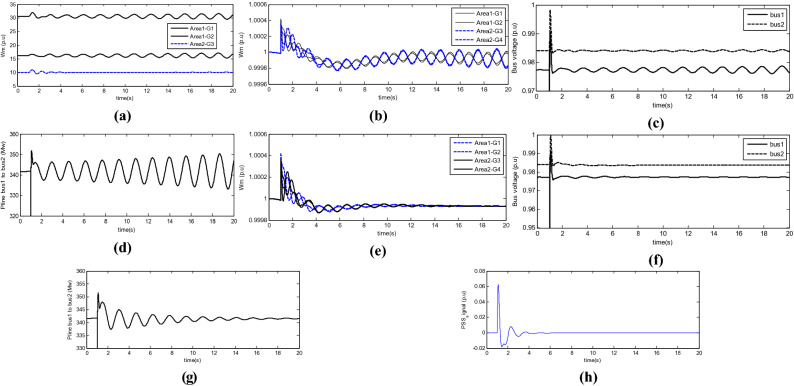
(**a**) Rotor angle of the turbines, (**b**) mechanical speed of each turbine rotor, (**c**) turbine connection bus voltage, and (**d**) inter-area transitional power oscillation and (**e**–**h**) are oscillations in turbine velocity.

To check the stability of the power system, 3—Phase-to-earth short circuit fault is placed in the middle of the line, which is released after a period. Figure 6 shows the rotor angle of the turbines, mechanical speed of each turbine rotor, turbine connection bus voltage, and inter-area transitional power oscillation.

**Figure 7:**
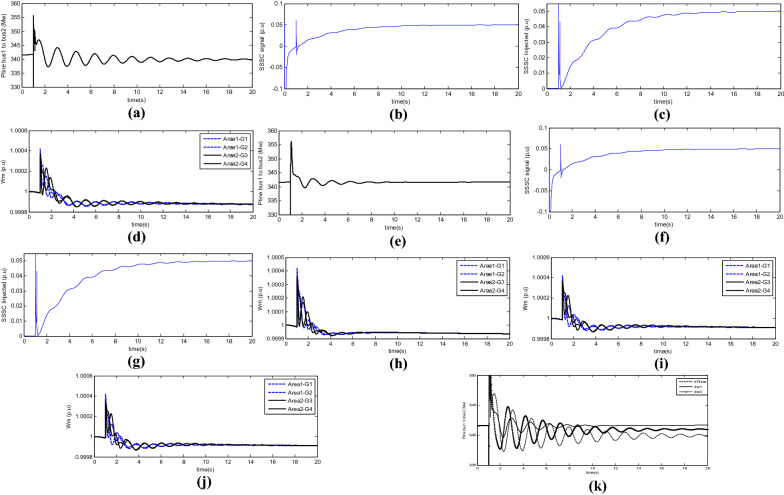
(**a**) Transient power oscillation signal, (**b**) signal generated by the proposed damper controller, (**c**) flow injected into line, (**d**) turbine velocity, (**e**) Transmit signal power fluctuation, (**f**) The signal generated by the proposed damping controller, (**g**) injected current to the line, (**h**) Generator speed, (**i**) Regional signal ${\Delta \omega }_{1}$ and (**j**) Regional signal ${\Delta \omega }_{2}$ and (**k**) power oscillation in the transmission line in dPline, *dw*_*1*_, and *dw*_*3*_ modes.

During a system failure, the system becomes subject to undamped oscillations. According to the oscillations in turbine velocity shown in the following Figure, the turbines in each area oscillate and divide the system into two areas. However, the unstable inter-area mode that is simulated and exists in the power system is visible in the transmission line power oscillations. These oscillations have 180 degrees of phase difference in the two areas, and their domain increases with time. The frequency of the inter-area oscillations in the two-area system is 0.7 Hz. In the first scenario, a traditional PSS power stabiliser is added to Area 2, and the system is then re-examined. The signals used in the PSS are ∆ω3 and ∆ω4. The values of the KSTAB, TW, T1, and T2 parameters are 10, 2.8, 0.024, and 0.34, respectively. The 3-Phase-to-earth short circuit fault is applied as before. In Fig. 7, the oscillations in turbine velocity are shown. The first and the last of the line bus voltages and the transmission power oscillations from the first to the second area are shown. Accordingly, the system is divided into two areas; however, due to the use of a synchronous PSS, the two areas are retained. In Fig. 6h, the signal of PSS applied to the system and the system excitation in the second area are illustrated. In the third scenario, an SSSC is added to the inter-area transmission line. The values of its parameters and the proposed parameters for the damper controller are listed in Tables 2 and 3, respectively.

**Table 2:** Parameters of the SSSC added to the transmission line.

Parameters	Value
Nominal voltage and frequency of transmission line	230 kV, 60 Hz
SSSC nominal power	100 MVA
Maximum voltage injection to transmission line	0.1 (p.u)
Series impedance converter	0.0053 + *j*0.16
Dc Link voltage	40kv
DC link capacitor	375 uF
PI controller coefficients for injection voltage	*K*_*p*_ = 0.00375 , *K*_*i*_ = 0.1875
PI controller coefficients for DC link voltage	*K*_*p*_ = 0.0001, *K*_*i*_ = 0.02

**Table 3:** Proposed parameters for the damper controller.

Parameters	Value
*K*_*stab*_	10
*T*_*w*_	2.8
*T*_*f*_	0.017
*T*_*1*_	0.075
*T*_*2*_	0.45

Firstly, to investigate the damping effect of different signals on inter-area oscillations, three signals; i.e., the transmission line oscillations, the velocity of the first area, and the velocity of the second area, were selected, and the simulation was performed using these three signals. The simulation was conducted using the dPline signal, which changes the inter-area transmission line power. The 3-Phase-to-earth short circuit fault is applied in the upper line. Figure 7a shows the transient power oscillation signal. In Fig. 7b, the signal generated by the proposed damper controller is shown.

Figure 7c shows the flow injected into the line according to the signal given by the damper controller, and Fig. 7d shows the turbine velocity. The area signals are used by the damper controller to generate the signal that *∆ω*_*1*_ used in Fig. 7e and the signal that *∆ω*_*3*_ used in Fig. 7g, h. To choose the most effective signal to improve damping, the results of these three signals are compared. Figure 7k shows the power oscillation in the transmission line in all three modes. The use of the signal indicating a change in turbine velocity has a greater effect on damping oscillations than the signal from the transmission line. Moreover, the first area signal has higher stability than the second area signal. According to these results, *∆ω*_*1*_ use has been used continuously.

#### Optimising SSSC parameters

For optimisation, firefly and harmony search algorithms are used. The parameters of each algorithm are shown in Table 4.

**Table 4:** Firefly algorithm parameters.

**Firefly algorithm**			
Optimization variables	nVar = 3	*K*_*stab*_*, T*_*1*_, *T*_*2*_	
Search space range	${K}_{stab}\in \left[1, 20\right]$	${T}_{1}\in \left[0.1, 1\right]$	${T}_{2}\in \left[0.01, 1\right]$
Maximum repetition	30		
Population	10		
$\alpha$	0.2		
${\beta }_{0}$	2		
$\gamma$	1		
**Harmony search algorithm**			
Optimization variables	nVar = 3	Kstab, T1, T2	
Search space range	${K}_{stab}\in \left[1, 20\right]$	${T}_{1}\in \left[0.1, 1\right]$	${T}_{2}\in \left[0.01, 1\right]$
Maximum repetition	30		
Population	6		
HMCR	0.9		

The optimisation variables include gain, time constant *T*_*1*_, and time constant *T2*. For each of the optimisation variables, a range of values is considered to cover all possible modes. On the other hand, due to the nature of each algorithm, suitable replications and populations were selected. Table 5 shows the results of optimising the two algorithms.

**Table 5:** Results of optimising the two algorithms.

Algorithm	*Kstab*	*T*_*1*_	*T*_*2*_
HS	14.74	0.71	0.39
FA	11.59	0.81	0.36

**Figure 8:**
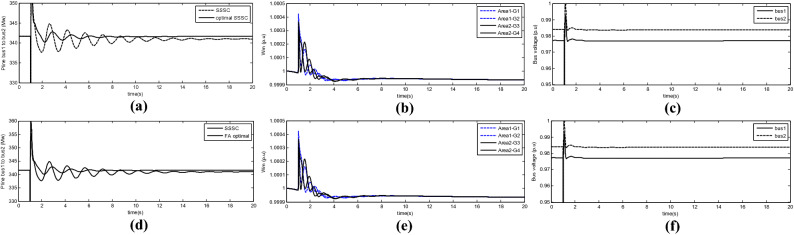
(**a**) Inter-area power oscillations using the SSSC optimised by the HS algorithm or the non-optimised SSSC, (**b**) Turbine rotor oscillations and (**c**) inter-area bus voltages, (**d**) Inter-area power oscillation using the SSSC optimised by the FA or the non-optimised SSSC, (**e**) turbine rotor oscillations with FA and (**f**) inter-area bus voltages with FA.

Figure 8a shows the inter-area power oscillations using the SSSC optimised by the HS algorithm or the non-optimised SSSC. Figure 8b indicates the turbine rotor oscillations and Fig. 8c shows the inter-area bus voltages.

Figure 8d shows the inter-area power oscillations using the SSSC optimised by the FA or the non-optimised SSSC. Figure 8e indicates turbine rotor oscillation and Fig. 8f shows the inter-area bus voltage. According to the results obtained from the two optimisation algorithms, the damping rate achieved by each one differs by 0.25%. The firefly algorithm outperformed the harmony search algorithm. Although the solutions produced with each algorithm differ little in performance, the calculation times for each algorithm differ significantly. Given the nature of the FA with the tested population and iterations, the time to obtain the optimal solution was 40 min. In contrast, the HS algorithm reached the optimal solution in significantly less time than FA. Table 6 shows the number of fitness function calls and the optimisation time for each algorithm.

**Table 6:** Number of calls and optimisation time.

Algorithm	Number of Fit Function Calls	Optimization time
FA	10 × 0 × 0 = 103	40 min
HS	6 × 31 = 186	17 min

## Conclusion

In this paper, the effect of SSSC on the small-signal stability of power system was investigated. The SSSC controller was designed and evaluated for damping power system oscillations. Firefly and harmony search algorithms were used to determine optimal parameters for the SSSC controller, and the capability of the optimised SSSC was evaluated. The effectiveness of two proposed algorithms and the degree of participation of SSSCs in damping inter-area oscillations was investigated. The phase constant of the phase compensator and its gain were considered as optimisation parameters. Determining the optimal compensator parameters will result in the minimum oscillation. To maximise system damping and minimise inter-area power oscillations, control signals from changes in the rotor angle of the turbines and changes in the power of the inter-area transmission line were used. The simulation was performed on a power system in two areas. The system turbines were modelled as a 6-order model, and the SSSC converter was modelled as an average model. The two-area system was then studied using PSS and SSSC power system stabilisers. According to the results obtained, the damping rate achieved by the two optimisation algorithms differs by 0.25%. The firefly algorithm outperformed the harmony search algorithm by 0.25%.
