# Author Correction: Optimal SSSC-based power damping inter-area oscillations using firefly and harmony search algorithms

**DOI:** 10.1038/s41598-020-72152-x

**Published:** 2020-09-18

**Authors:** Amirreza Naderipour, Zulkurnain Abdul‑Malek, Vigna K. Ramachandaramurthy, Mohammad Reza Miveh, Mohammad Jafar Hadidian Moghaddam, Josep. M. Guerrero

**Affiliations:** 1 Institute of Research and Development, Duy Tan University, Da Nang, 550000 Vietnam; 2 Faculty of Electrical Electronic Engineering, Duy Tan University, Da Nang, 550000 Vietnam; 3 Institute of High Voltage & High Current, School of Electrical Engineering, Faculty of Engineering, Universiti Teknologi Malaysia, 81310 Johor Bahru, Malaysia; 4 Institute of Power Engineering, Department of Electrical Power Engineering, College of Engineering, Universiti Tenaga Nasional, Jalan Ikram-Uniten, 43000 Kajang, Malaysia; 5 Department of Electrical Engineering, Tafresh University, 39518‑79611 Tafresh, Iran; 6 College of Engineering and Science, Victoria University, Melbourne, 3047 Australia; 7 Institute of Energy Technology, Aalborg University, Aalborg East, Alboorg, Denmark

Correction to: *Scientific Reports* https://doi.org/10.1038/s41598-020-69123-7, published online 22 July 2020

The Acknowledgements section in this Article is incorrect. 

“This research was funded by the Universitas Sriwijaya (grant 4B379), Universiti Malaysia Perlis (4B482), Universiti Teknologi Malaysia (Post-Doctoral Fellowship Scheme grant 05E09, and RUG grants 01M44, 02M18, 05G88) and VILLUM FONDEN under the VILLUM Investigator Grant (no. 25920): Center for Research on Microgrids (CROM); https://www.crom.et.aau.dk. In addition, the authors wish to thank Duy Tan University for their financial support.”

should read:

“This research was funded by the Universiti Teknologi Malaysia (Post-Doctoral Fellowship Scheme grant 05E09), and VILLUM FONDEN under the VILLUM Investigator Grant (no. 25920): Center for Research on Microgrids (CROM); www.crom.et.aau.dk. In addition, authors would like to acknowledge the funding from iRMC Internal Research Grant (RJO10517844094), Universiti Tenaga Nasional, Malaysia.”

